# Nutritional Care in Children with Cystic Fibrosis

**DOI:** 10.3390/nu15030479

**Published:** 2023-01-17

**Authors:** Elena Mariotti Zani, Roberto Grandinetti, Daniela Cunico, Lisa Torelli, Valentina Fainardi, Giovanna Pisi, Susanna Esposito

**Affiliations:** Pediatric Clinic, Department of Medicine and Surgery, University of Parma, 43126 Parma, Italy

**Keywords:** CFTR modulator, cystic fibrosis, macronutrients, malnutrition, nutrition, pancreatic enzyme replacement therapy, vitamin

## Abstract

Patients with cystic fibrosis (CF) are prone to malnutrition and growth failure, mostly due to malabsorption caused by the derangement in the chloride transport across epithelial surfaces. Thus, optimal nutritional care and support should be an integral part of the management of the disease, with the aim of ameliorating clinical outcomes and life expectancy. In this report, we analyzed the nutrition support across the different ages, in patients with CF, with a focus on the relationships with growth, nutritional status, disease outcomes and the use of the CF transmembrane conductance regulator (CFTR) modulators. The nutrition support goal in CF care should begin as early as possible after diagnosis and include the achievement of an optimal nutritional status to support the growth stages and puberty development in children, that will further support the maintenance of an optimal nutritional status in adult life. The cornerstone of nutrition in patients with CF is a high calorie, high-fat diet, in conjunction with a better control of malabsorption due to pancreatic enzyme replacement therapy, and attention to the adequate supplementation of fat-soluble vitamins. When the oral caloric intake is not enough for reaching the anthropometric nutritional goals, supplemental enteral feeding should be initiated to improve growth and the nutritional status. In the last decade, the therapeutic possibilities towards CF have grown in a consistent way. The positive effects of CFTR modulators on nutritional status mainly consist in the improvement in weight gain and BMI, both in children and adults, and in an amelioration in terms of the pulmonary function and reduction of exacerbations. Several challenges need to be overcome with the development of new drugs, to transform CF from a fatal disease to a treatable chronic disease with specialized multidisciplinary care.

## 1. Introduction

Cystic fibrosis (CF) is a progressive genetic disease that causes persistent lung infections and limits the ability to breathe over time [1]. CF is also the most common life-shortening autosomal recessive disease [2,3]. CF is caused by mutations in the cystic fibrosis transmembrane conductance regulator (CFTR) gene, located at 7q31.2, which encodes the CFTR protein. This mutation leads to the absent or decreased function of the CFTR protein at the cell surface [4]. Currently, over 1900 CFTR mutations have been identified, the most common being a deletion at position 508, annotated as F508del [5,6,7]. Since specific mutations cause dysfunctions in the CFTR protein in various ways, six different classes have been defined to categorize the nature of the protein’s dysfunction. Classes I, II and III (defective protein production, defective processing and defective regulation) have more severe disease phenotypes and are more strongly associated with pancreatic insufficiency (PI) [5,6,7]. Indeed, the lack of the adequate functioning of the CFTR protein causes a disruption of the transport of sodium and chloride ions across cell membranes, leading to thickened mucus secretions in organs throughout the body, including the lungs, liver, pancreas, gallbladder and intestines [8,9,10]. In the lungs, thickened mucus adheres to the airway surfaces, which leads to a reduction of the mucociliary clearance, and to an increased risk of inflammation and infection. In the pancreas, thickened secretions obstruct the intra-pancreatic ducts, reducing the delivery of digestive enzymes to the intestines and impairing the absorption of key nutrients [10]. Intestinal malabsorption, in combination with an increased metabolic demand to achieve homeostasis, leads to challenges in providing an adequate nutritional intake. Thus, CF patients are prone to malnutrition, vitamin deficiencies and poor growth, in addition to the pulmonary manifestations [11,12]. For this reason, nutritional management is a fundamental part of the multidisciplinary approach of the patient with CF, at all ages [13,14]. The target of nutritional management is to reach and maintain an optimal nutritional status; advances in both pulmonary and nutritional management reached in recent years have helped to improve the nutritional status of patients with CF [13,14].

Newborn screening has had a profound impact on nutrition outcomes. As a matter of fact, infants diagnosed with CF via newborn screening, and children introduced to nutritional therapy at an earlier age, have a greater growth than those diagnosed later in infancy and childhood [15]. In this narrative review of the literature, we analyzed the current evidence in the field of CF and nutrition in children. This review was carried out by the Emilia-Romagna Regional Center for Cystic Fibrosis at the Pediatric Clinic at the University of Parma, Parma, Italy. Systematic searches were performed in PubMed, Cochrane Library, Google Scholar and ClinicalTrials.gov up, to July 2022. Language was restricted to English. Search terms included cystic fibrosis (CF), nutrition, enteral nutrition, parenteral nutrition and CFTR modulators, in combination with neonate, newborn, infant and preterm infant. Case reports, case series, original research studies, review articles, letters to the editor, randomized controlled trials (RCTs), non-RCTs and cohort studies (prospective or retrospective) were included.

## 2. Pathogenesis of Malnutrition in Cystic Fibrosis

There is a strong association between nutritional status and clinical outcomes in CF patients. In children with CF, malnutrition and poor lung function are the main sources of morbidity. Previous studies found a link between fat absorption and pulmonary outcomes: children who had steatorrhea had more severe symptoms, a higher sweat chloride and an overall worse prognosis than children who had a normal fat absorption [15,16]. The respiratory status and survival in children and adults inversely correlate with the degree of malnutrition, including short stature, lower weight and body mass index (BMI) [17,18]. Lung function at older ages is also associated with the nutritional status in toddlerhood and childhood [19,20,21,22]. Ashkenazi et al. found that a BMI z-score < −0.75 at 10 years of age was associated with a higher rate of lung transplantation in adulthood [22].

One of the first systems that shows symptoms of CF-related disease is often the gastrointestinal tract (GI). The GI symptoms, in fact, can be present even in utero or precede the pulmonary symptoms. In the association between malnutrition and CF, several factors are involved: a lower energy intake, greater energy expenditure, higher essential fatty acid (EFA) turnover, endocrine and exocrine PI, enteric inflammation, bacterial overgrowth and impaired bicarbonate secretion [23,24,25,26,27,28]. Multiple GI tract abnormalities are involved as the cause of malabsorption, which is a multifactorial process. In patients with CF, the primary mechanism of malabsorption is the PI. Approximately 85% of patients with CF develop PI by the age of one [29]. The ductal obstruction is predisposed by the thickened secretions caused by impaired chloride and bicarbonate secretion. The obstructions in the pancreatic ducts damage the transport of the digestive enzymes and pancreatic bicarbonate to the intestinal lumen. This leads to exocrine PI, poor absorption of fat-soluble vitamins (A, D, E and K) and acidifies intestinal lumen [7,9,11]. This process could begin in utero and progress through the loss of acinar tissue, ductal fibrosis and disruption and, eventually, lead to the fatty transformation of pancreatic tissue. As mentioned before, the severity of the CFTR variant correlates with PI: patients with three severe variants (classes I, II and III) have an altered exocrine function early in life, often at birth, while patients with two mild CFTR variants (classes IV and V) or who are heterozygous for mild/severe variants are usually pancreatic sufficient (PS) at birth [30].

In patients with CF, intestinal acidification also contributes to malabsorption. The progressive destruction of the pancreas deprives the duodenum of pancreatic secretions rich in bicarbonate [31]. Multiple deleterious effects on absorption can result from an unbuffered intestinal acidification: first, lipase is irreversibly inactivated at a pH below 4.0 [31,32]; second, the coatings of most commercial pancreatic enzyme replacement therapy (PERT) preparations are resistant to degradation at an acidic pH; third, at a pH below 5.0, glycine-conjugated bile acids precipitate out of an aqueous solution, resulting in an impaired bile acid-mediated micellar formation and lipid absorption, which makes fat absorption very difficult [33].

Patients with CF can also have an abnormal motility throughout the GI tract [34]. The reduction of enteral consumption and thus nutrient and caloric intake, caused by poor motility, can impact nutrition. With regards to food consumed by mouth as well as by tube, the delayed gastric emptying in the stomach can lead to early satiety and limit enteral intake. In addition, delayed gastric emptying can be exacerbated by the higher fat diet recommended for CF patients. Because of this, interventions for delayed gastric emptying, such as prokinetic agents, should be considered on an individual basis. In patients with CF, the small bowel transit time is also delayed, but the clinical significance of this, as a pathogenetic factor of malnutrition, is unknown at this time [34]. One set of views proposes that thickened CF intestinal mucus overlies the epithelium and leads to an abnormal chyme-epithelium interface, causing the decreased absorption of nutrients [9,35,36]. Another hypothesis proposes that the abnormally thickened intestinal mucus causes the stasis of intestinal contents and serves as a biofilm, the combination of which leads to dysbiosis and frequent small bowel bacterial overgrowth (SBBO) [35,37,38]. Other studies have hypothesized, based on experiments on CF-mutant mouse models, that the intestinal smooth muscle itself develops dysmotility [39,40,41]. Even though the mechanism is not entirely clarified, it does not seem to be a direct effect of CFTR, but more likely related to a complex network involving a different expression in prostaglandins, possibly influenced by dysbiosis [39,40,41]. In the intestines of patients with CF, Brunner’s gland also presents an altered functioning, particularly where CFTR is highly expressed [9,37].

Furthermore, approximately 40% of people with CF are affected by gastroesophageal reflux disease (GERD), which is the most common GI complication of CF [42]. Dyspepsia, dysphagia, acid brush, heartburn and chest pain, which are the clinical symptoms for GERD, and a decreased appetite can limit food intake. Because of this, it is important to remember and recognize GERD as an important cause of malnutrition in people with CF [42]. Acid suppressors, used by clinicians for the treatment of GERD, are also used to potentiate the intestinal acidification, in an attempt to improve the PERT function and thus the absorption of nutrients [42,43].

Moreover, there are significant nutritional implications of CF-related liver disease (CFRLD), of which there are many different types [44]. Especially neonatal cholestasis, which is often the initial sign of CFRLD, affects nutrition. Cholestasis in CFRLD is likely due to the increased viscosity of the bile from CFRLD dysfunction [44]. Cholestasis, besides determining parenchymal inflammation and fibrosis through the reduction of the total bile acid content and overall concentration of bile in small intestines, also limits the amount of bile acids available for digestion. Because bile acids are required for lipase function, neonatal cholestasis can exacerbate fat malabsorption. All of these mechanisms can lead to a deficiency in fat-soluble vitamins, which is more severe than in patients without neonatal cholestasis [44].

Overall, these factors cause the marked malabsorption of nutrients and bile acids, the latter of which further contributes to a poor absorption of the fats and fat-soluble vitamins [37]. Moreover, the bile acid profile is associated with the severity of liver disease, with glycodeoxycholic acid (GDCA), which allows us to differentiate patients with non-cirrhotic liver involvement from those with no detectable liver disease [45]. Furthermore, the disorder of the fatty acid profile deepens in patients with liver diseases and cirrhosis and leads to other disorders of lipid metabolism and dyslipidemia [46,47,48,49]. Patients with CF have a reduced intestinal absorption of cholesterol and plasma sterol profile. It may be useful to evaluate the metabolic status of lipids of patients with CF, in order to help manage the pancreatic enzyme supplementation therapy [47].

Clearly, interventions to improve the nutritional status, such as supplemental feeding (enteral and parenteral), PERT and behavioral interventions to increase calories, have a positive impact on weight and consequently on the respiratory status [50,51,52,53,54,55,56,57,58,59]. The introduction of neonatal screening leads to an improvement in long-term growth, in a reduction of pulmonary exacerbations and in an increase in survival, thanks to an earlier introduction of nutrition intervention [15,60,61,62,63].

## 3. Nutritional Assessment in Cystic Fibrosis

As mentioned before, evaluation and management of the nutritional status of patients with CF have an impact on their clinical outcomes [10]. A correct evaluation begins with newborn screening. Indeed, this practice is supported by evidence that an early diagnosis and then prompt intervention are effective in determining a better quality of life and in improving the lung function and growth parameters [60,64,65]. Worldwide, there are different screening protocols for CF, all implying a first dosage of immunoreactive trypsinogen (IRT) on a newborn blood spot, then variably linked to an IRT repeat measurement, a CFTR mutations analysis, pancreatitis associated protein (PAP) evaluation and sweat test for chloride concentration [66,67].

### 3.1. Growth Parameters

Once CF is diagnosed, the assessment of anthropometric parameters reflecting the nutritional status are recommended as part of routine CF care, with the aim of achieving a nutritional condition comparable to that of healthy children [10]. The reference parameters utilized are different, depending on age and basal nutritional status [43]. For infants and children younger than two years of age, weight, height and weight-for-length percentiles should be used to evaluate growth [10,68]. The longitudinal growth trajectory should also be used as a benchmark [69]. A weight-for-length percentile equal to or greater than the 50th percentile, according to the Centers for Disease Control (CDC) growth charts [70], is recommended [43,71]. Since CF patients achieving a 50th percentile of the weight-for-length, according to the World Health Organization’s (WHO) definition, have been found to have a lower forced expiratory volume (FEV1) than CF patients at the 50th percentile. Naturally, use of the latest charts are preferred [43].

In children from two to 18 years of age, the nutritional status may be assessed by the BMI [10], with the recommendation from the CDC charts to attain and maintain a BMI percentile equal to or greater than 50, [72]. A BMI at or above 22 kg/m^2^ and 23 kg/m^2^ is recommended for females and males, respectively [10].

The Cystic Fibrosis Foundation (CFF) guidelines recommend performing monthly anthropometric parameter assessments up to six months of life and then every one to two months from six months to one year of life [68]. However, it is essential to focus special attention to the nutritional status during the first 12 months after the diagnosis of CF, throughout both the first year of life and the prepuberty period. [68]. So, ESPEN-ESPGHAN guidelines suggest growth clinic monitoring every 1 to 2 weeks, then monthly through the first twelve months of life [10]. Older children and adults are suggested to be assessed at least every 3 months. Patients who fail to meet these criteria for length and weight should have more frequent monitoring and management [43].

### 3.2. Laboratory Tests and Other Measures

ESPEN-ESPGHAN guidelines also recommend the annual monitoring of the biochemical markers of malnutrition, including blood count, iron status, plasma fat-soluble vitamin levels, serum liver function and electrolyte measurements [10]. The annual determination of the coagulation factors should be advisable, given the possibility of a vitamin K deficiency [43]. A yearly fecal pancreatic elastase-1 dosage should be assessed, in order to exclude an exocrine pancreatic insufficiency [10].

In addition to the BMI, the assessment of the body composition should be considered, with the aim of reaching the target BMI, by increasing muscle mass instead of fat mass [72]. Since the evidence shows that the BMI is less strongly associated with lung function and nutritional status, compared to lean body mass (LBM) and bone mineral content (BMC) [73], the guidelines propose the measurement of the composition of the body weight from the age of eight to 10, every one to five years [10]. Different techniques could be utilized, including dual-energy X-ray absorptiometry (DXA), anthropometry or bioelectrical impedance [10].

All CF patients should undergo oral glucose tolerance testing yearly, beginning from 10 years of age [10,74]. Part of the nutrition status evaluation should be a dietary review, at least every 3 months for children and adolescents, using questions about the adherence to dietary advice and a record of dietary habits 3 to 5 days long [10].

## 4. Energy Requirements

The standard of care for patients with CF is a higher caloric intake than healthy subjects, as they are susceptible to an energy deficit, due to an imbalance between the energy intake and energy losses and requirements [43]. Energy losses in CF are greater than those in the normal controls. Intestinal malabsorption, even if pancreatic enzyme supplementation seems to control the clinical signs and symptoms, leads to energy losses through steatorrhea and azotorrhea [75,76]. Furthermore, type 2 diabetes mellitus may contribute to energy losses through glucosuria, as well as protein loss due to sputum [75,76]. Lastly, fat malabsorption could be the consequence of an inadequate bile salt secretion due to liver cirrhosis [75].

Another parameter in determining energy requirements is the measure of energy expenditure. Resting energy expenditure (REE) is responsible for 60–70% of the total energy expenditure, while the 10–25% is attributable to daily physical activity and the remaining 10% to diet-induced thermogenesis [76]. The REE is increased by 10–30%. The severity of lung disease contributes to the rising REE, especially because of the higher workload for the respiratory muscles [77]. During a pulmonary exacerbation, the REE becomes approximately twice as high as the normal controls, turning to lower values after treatment [76,77]. Furthermore, a pancreatic insufficiency has been linked to an increased REE [78]. The latter may also be mediated by catecholamines and cytokines during chronic inflammation [77]. Salbutamol and other beta-adrenergic drugs have been reported to increase the REE during the first hour after inhalation [76].

The energy intake, evaluated on the pattern of weight loss or gain and of adipose stores, is usually inadequate in patients with CF [68]. Compensatory digestive mechanisms, such as salivary amylase, gastric lipase, pepsin and colonic microbiota, are effective in releasing energy from food. However, these alternative mechanisms are less efficient than the usual ones [10]. Respiratory impairment, vomiting and clinical depression may cause anorexia [75]. Other conditions that could lead to a decrease in appetite and thus in the energy intake in CF patients are intestinal obstruction, gastroesophageal reflux and the release of cytokines in a condition of chronic inflammation or during pulmonary exacerbation [75].

Due to the pathogenesis of the disease and of the malabsorption, and because of the energy deficit, the standard of care for patients with CF is a high-calorie and high-fat diet with pancreatic enzyme replacement therapy (PERT) and the oral supplementation of vitamins [69].

There is a relevant variability between individuals with CF, regarding the energy daily requirement, depending on the degree of malabsorption, the level of chronic inflammation, lung function and exacerbations. The energy intake recommended by the European guidelines range from 120–150% concerning the energy requirements of the healthy population of similar age, sex and size [10]. Similarly, US guidelines recommend a higher dietary intake from 110% to 200% for the energy requirements of the healthy population [71].

## 5. Macronutrients: Fats, Proteins, Carbohydrates in the Diet of Patients with Cystic Fibrosis

The European and American consensus guidelines recommend that CF children receive over 120% of their energy requirements, according to age [79,80]. Because of the pathogenesis of the disease, as explained before, the decreased availability of lipase and bile salts negatively influences the digestion and absorption of fats. Overall, 35 to 40% of calories in a CF diet are obtained through fats (that is a greater intake than that recommended for healthy people) [68].

Furthermore, the protein intake should be higher for CF patients than the general population, so as to avoid protein malnutrition, identified by low serum albumin levels [74]. In the diet of patients with CF, proteins account for approximately 15 to 20% of calories [68]. Although it is recommended that 20% of the calories should come from protein, the optimal protein needs of CF patients are likely to be much higher, as indicated in other chronic inflammatory disease states [80,81]. The optimal protein intake of CF patients is crucial to prevent muscle loss [82,83].

Given the recommendations for an increased fat and protein intake, the resulting carbohydrate goal is lower than for the general population: if patients are following a high calorie diet, this carbohydrate intake is not typically difficult to achieve and a specific goal does not need to be set [69]. Carbohydrate provision must be adjusted according to the calories requirements but also according to the presence or absence of impaired glucose tolerance or CF-related diabetes [84]. In fact, for people with CF and diabetes, careful attention must be paid to multiple aspects, such as glycemic control and energy requirement adequacy, as these are linked with the risk of metabolic impairment and cardiovascular disease [1]. The prominent scientific societies dealing with these aspects (American Diabetes Association, Cystic Fibrosis Foundation, Pediatric Endocrine Society) provide important guidance recommending [10]:

- Individualized carbohydrate intake that is monitored for glycemic control;
- A higher than standard intake of calories and protein;
- A high fat diet, as needed, for weight maintenance and for the compensation of essential fatty acids;
- A limited use of artificial sweeteners due to the need for adequate calories.

Patients with CF, especially if pancreatic insufficiency is present, might undergo abnormalities in serum fatty acids and in essential fatty acid (EFA) deficiency [69]. The etiology is multifactorial: the decreased lipolysis can cause a poor absorption in long-chain fats; the inflammatory cascade leads to a raised turnover; both the high oxidative stress due to infections and the negative energy balance caused by a low weight cause the destruction of polyunsaturated fatty acids [69,76]. EFA deficiency, evaluated by measuring the level of linoleic acid or the triene:tetraene (T3:T4) ratio [85], can involve clinical manifestations, such as gastrointestinal or pulmonary symptoms, hepatic, renal, immune dysfunctions, desquamating skin lesions, growth retardation and increased susceptibility to infections [69,75]. The severe CF phenotypes are associated with a deficiency of linoleic acid, which is the precursor of arachidonic acid [86]. The release of arachidonic acid from membranes via phospholipase A_2_ is the rate-limiting step for eicosanoid synthesis and is increased in CF, which contributes to the observed inflammation. A potential deficiency of docosahexaenoic acid may lead to decreased levels of specialized pro-resolving mediators. This pathophysiology may contribute to an early and sterile inflammation, mucus production, and to bacterial colonization, which further increases inflammation and potentiates the clinical symptoms [86].

EFA supplementation seems to determine the anti-inflammatory effects and to improve lung function, also decreasing the number of exacerbations and the duration of intravenous antibiotic therapy [87]. Thus, the guidelines for the correct prescription of foods for special medical purposes for CF patients, according to the Italian Ministry of Health, suggest supplementation with docosahexaenoic acid (DHA) at the dosage of 100 mg/Kg/day in the first year of life [88]. Nevertheless, current international guidelines cannot recommend the dietary supplementation of fatty acids, based on the available evidence. Patients with CF should receive an intake of fatty acids, as recommended for the general population [10,68,76].

## 6. Fat-Soluble Vitamins

A deficiency in fat-soluble vitamins is common in people with CF, which is essentially due to the altered mechanism of fat absorption and reduced luminal bile salts [69]. Furthermore, patients with pancreatic insufficiency are at risk of a fat-soluble vitamin deficiency, despite enzyme replacement [89]. Liposomes can enhance the bioavailability of vitamin A, and cyclodextrins may strengthen the supplementation of vitamins D3 and E relative to the medium-chain triglycerides (MCT) in pancreatic-insufficient CF [90].

### 6.1. Vitamin A

Low levels of vitamin A are observed in patients with CF, because of a pancreatic insufficiency, which predisposes them to the malabsorption of fat and fat-soluble vitamins [91]. Vitamin A is important for vision, epithelial differentiation and resistance to infectious diseases, as well as for the antioxidant properties of its precursor, beta-carotene [92]. Thus, such a deficiency may have clinical consequences even if exceptional, such as night blindness, xerophthalmia and abnormalities of the bronchial mucosal epithelium [75].

Furthermore, it has been studied that low serum levels of vitamin A are associated with a poorer clinical status, impaired lung function, and increased pulmonary exacerbations [93]. It can be related to the systemic inflammation status. Indeed, during the acute phase response, the mobilization of retinoids from hepatic cells is impaired, as well as the retinol binding protein (RBP) [69,92]. In addition, the requirement of vitamin A may be increased by the oxidative stress linked to inflammation [92]. However, until an optimal range for serum retinol levels in CF has been defined, clinicians should aim for the minimum vitamin A supplementation dosage necessary to reach sufficient serum retinol levels to prevent deficiency and avoid hypervitaminosis A, which could lead to bone disease and liver damage [91].

The ESPGHAN guidelines affirm that in CF patients with PI, a daily provitamin beta carotene dose of 1 mg/kg body weight/day for 12 weeks, followed by a maintenance dose (maximum of 10 mg/day) was found to be efficacious and safe for children aged six to 18 years [10,91,94]. The administration of beta-carotene, i.e., a precursor of vitamin A, is considered safer than retinol because it is subject to negative feedback control [95,96].

### 6.2. Vitamin D

Vitamin D is an important mediator of immunity and respiratory health [97]. This hormone, as determined by circulating 25-hydroxyvitamin D 25(OH)D concentrations, upregulates the production of an antimicrobial peptide, cathelicidin, in in vitro and in clinical studies [98,99], providing strong immunomodulatory effects.

A vitamin D deficiency is a common finding in patients with CF. Despite supplementation, most of the patients remain at risk for a vitamin D deficiency due to fat malabsorption that is secondary to an exocrine pancreatic insufficiency, decreased sunlight exposure, poor nutritional intake, and changes in the vitamin D metabolism [100,101].

Several studies have documented that a vitamin D deficiency increases the risk of pulmonary exacerbation in children and adults with CF [102,103]. Screening for vitamin D deficiency and the appropriate vitamin D supplementation ensures an adequate vitamin D status, which has been positively associated with a higher bone density in children and adults with CF and with improved recovery after a pulmonary exacerbation of CF [104,105].

The European cystic fibrosis bone mineralization guidelines recommend a minimum serum 25(OH)D threshold of 20 ng/mL and the US Cystic Fibrosis Foundation suggests that individuals have sufficient vitamin D levels if 25(OH)D levels are >30 ng/mL [106,107]. The ESPEN-ESPGHAN-ECFS guidelines on nutrition care for infants, children, and adults with cystic fibrosis recommends starting infants on D3 (cholecalciferol) at a dose of 400 IU/day (advance to upper limit of 1000 IU/day) [10]. The monitoring of serum 25 (OH) D levels has to be carried out annually or checked 3 to 6 months after a dosage change [10].

### 6.3. Vitamin K

A vitamin K deficiency is well-recognized in people with CF and PI [108]. In addition to the disease process of CF and the insufficient supplementation, another co-factor that contributes to determining low vitamin K levels is the long-term use of certain types of antibiotics, which modify the intestinal flora which produces vitamin K [109]. The manifestations of a vitamin K deficiency can range from a mild subclinical identification (e.g., low levels of vitamin K in the blood) to actual coagulopathy. Such manifestations may include gastro-intestinal bleeding, hematuria, epistaxis and subcutaneous bleeding. Vitamin K is also involved in the calcium binding proteins in the bone and its deficiency is implicated in defective bone remineralization and reduced bone mineral density (BMD) and thus osteopenia and osteoporosis [109]. Vitamin K-related carboxylation allows for the activation of the bone matrix protein, osteocalcin, resulting in the osteoblast function and bone formation; a vitamin K deficiency impairs this process and thus impairs bone formation [110].

Currently, there are no routinely used biomarkers for the vitamin K status. It can be evaluated by measuring serum concentrations of vitamin K, PIVKA-II (protein induced by vitamin K absence) and undercarboxylated osteocalcin, but these biochemical indicators are not commonly used in routine clinical practice due to their cost [10]. The prothrombin time can be measured but the results will recognize only a severe deficiency in vitamin K [10].

Dougherty et al. affirmed that in children and young adults with CF and PI, vitamin K levels are often suboptimal despite routine supplementation with CF-specific preparations. Only subjects taking high doses of vitamin K (1000 lg/d) achieved a vitamin K status similar to that of healthy subjects [111]. Collectively, these findings suggest that the routine supplementation of high doses of vitamin K for this population will be required to ensure an optimal response [111]. Another study conducted by Beker et al. supports the daily, rather than weekly, supplementation of vitamin K [112] because of its limited storage capacity and rapid metabolic turnover [113]. ESPGHAN guidelines suggest a regular supplementation of vitamin K at 0.3 to 1.0 mg/day for infants [106]. For older children and adults, ESPGHAN suggests 1 to 10 mg/day of vitamin K [106,111]. In the case of sustained antibiotic therapies, higher doses may be considered [108,111].

### 6.4. Vitamin E

Vitamin E is a generic term for a group of eight fat-soluble compounds, the tocopherols and tocotrienols, of which α-tocopherol has the main biological activity [114]. It functions as an antioxidant that protects cell membranes from oxidative damage [115]; its deficiency may worsen the burden of oxidative stress that results from constant inflammation, especially in respiratory and digestive systems which occurs in CF [116].

Low vitamin E levels in CF patients put them at an increased risk of the detrimental effects of a vitamin E deficiency [117]. Vitamin E deficiency disorders are rare events and include cerebellar ataxia, peripheral neuropathy, generalized weakness, myopathy, pigmented retinopathy and visual field contrition with loss of vision [118]. A vitamin E deficiency can also manifest as the cognitive impairment and hemolytic anemias because of its role in the maintenance of the structural integrity of the hemoglobin membrane [119,120,121]. The risk of a vitamin E deficiency increases with the inflammation of the respiratory or digestive system [93,116]. Since vitamin E (α-tocopherol) is transported almost exclusively in circulating lipoproteins in blood, serum levels depend strongly on the amount of circulating lipids, which are often low in patients with CF [122]. It may be more accurate to use the α-tocopherol:total lipid ratio (in fasting samples), or the α-tocopherol:cholesterol or α-tocopherol:polyunsaturated fatty acids ratios (in non-fasting samples). A recent study conducted by Sommerburg et al. investigated the concentration of vitamins A and E in children and adolescents with CF homozygous for the CFTR mutation Phe508del before and after at least 12 months of treatment with lumacaftor and ivacaftor [122]. The authors observed a slight decrease of the plasma vitamin E level and explained that there may be various reasons for this. One of these could be that vitamin E competes with other fat-soluble substances when absorbed from the intestine. In addition, CFTR modulators normalize the intestinal pH, which is also expected to alter the fat digestion in the intestine [123].

Another reason for a slightly decreased plasma vitamin E concentration could be an altered vitamin E distribution in the adipose tissues of CF patients, where vitamin E is accumulated to a considerable extent [124]. A further reason may be due to lumacaftor itself, as this is a known inducer of cytochrome P450 3A4, which in turn would also affect the metabolism of vitamin E [124,125].

The European guideline on nutrition for children and adults with CF recommends a regular supplementation of vitamin E with 100 to 400 IU/day, 50 IU/day for infants < 12 months to maintain the serum α-tocopherol:cholesterol ratio above 5.4 mg/g [10,126]. The monitoring of vitamin E levels has to be carried out annually or checked 3 to 6 months after a dosage change [10].

## 7. Electrolytes and Minerals

As a consequence of the increased sweating, intestinal malabsorption, and chronic inflammation, patients with CF may have higher than normal requirements for electrolytes and minerals [10].

### 7.1. Sodium

CF pediatric patients may be at an increased risk of sodium depletion, which may lead to impaired growth and can trigger electrolyte disturbances, especially when it occurs in infants, because of their low sodium containing diet (breastmilk < 7 mmol/L and infant formula < 15 mmol/L), the larger relative body surface area, and immature homeostatic mechanism [10,127,128,129,130]. This may lead to chronic hyperaldosteronism that is secondary to the increased renin release [131,132].

Nutrition guidelines for CF management report variable recommendations for salt intake and supplementation. According to the guidelines of the Cystic Fibrosis Foundation, routine salt supplementation is recommended [130], whereas the ESPGHAN guidelines recommend evaluating and supplementing when needed [10]. In the absence of fever, hot weather, or exercise, a Western diet is assumed to contain a sufficient amount of salt to compensate for the sodium-chloride needs, especially in older children and adults [10].

A useful non-invasive measure of the adequacy of sodium supplementation in CF pediatric patients is the serum sodium concentration and/or urinary sodium, as a marker for the sodium status [133,134,135,136].

### 7.2. Iron

Children with CF are at high risk of iron deficiency (ID) [137]. Chronic inflammation and the malabsorption of iron are secondary to pancreatic insufficiency, and malnutrition and an inadequate intake are often seen in CF, making these patients particularly susceptible to ID. The incidence of ID in patients with CF is estimated from 33% in the pediatric population [137] to over 60% in adult patients [138].

The WHO defines ID as a ferritin concentration below 12 mg/L in children under five years of age and below 15 mg/L in children over five years of age [139]. Conventional markers of iron status are often abnormal in patients with CF, reflecting inflammation and/or infection, rather than the actual iron deficiency. Ferritin, for instance, is an acute phase protein, so higher values may indicate the severity of inflammation rather than improving the iron status [140]. The soluble transferrin receptor (sTfR) was the best parameter in assessing iron deficiency in CF. However, Uijterschout et al. demonstrated that sTfR is not useful in diagnosing iron deficiency in healthy children with CF [141].

ESPGHAN guidelines suggest monitoring children, adolescents, and adult patients annually using serum iron determination and, in cases of ID, recommend resolving the underlying inflammation, and supplementing with iron only if deficiency persists [10,68].

### 7.3. Calcium

Decreased bone mineral density is commonly found in CF patients [142,143,144]. Potential risk factors include chronic inflammation, gastrointestinal malabsorption, pulmonary infection, pancreatic insufficiency, vitamin D and K deficiencies, negative calcium balance, hypogonadism and CF-related diabetes, and these require prolonged glucocorticosteroid treatment and immunosuppressive therapy [145,146]. Decreased bone mineral density may contribute to a higher risk of low-trauma fractures that make chest physiotherapy ineffective and accelerate the deterioration of the lung function [147,148].

ESPGHAN guidelines recommend an annual assessment of the calcium intake even if currently, a simple test of the calcium status is not available. Calcium-rich foods, such as dairy foods, should be encouraged, especially in patients with a suboptimal calcium intake, to prevent bone demineralization [10].

### 7.4. Zinc

Zinc is an essential trace element that participates in many metabolic processes as a catalytic, regulatory and structural component [149]. Zinc-dependent proteins play roles in transcriptional regulation processes, DNA repair, apoptosis, extracellular matrix regulation and antioxidant defense [150]. Zinc is important for human health and disease due to its critical roles in growth and development, bone metabolism, central nervous system, immune function and healing processes [151,152].

A zinc deficiency impairs the specific susceptibility to bacterial, viral and fungal pathogens [153]. This triggers numerous health problems in children with CF, such as weight loss, stunted growth, weakened resistance to infections and early death [154]. The clinical signs of a marginal zinc deficiency include a decrease in immunity, taste and smell senses, night blindness, memory impairment and sterility [155]. Hypozincemia, assessed with a laboratory cut-off of 40 mg/dL, has been reported in approximately 30% of young infants with CF, during newborns screenings [156], Maqbool et al. reveal a prevalence of low plasma zinc ranging from 0% to 40% in various populations of infants, children and adolescents with CF [157].

CF patients may benefit from zinc supplements in several different aspects of their disease. A retrospective analysis of clinical data suggests that zinc supplements improved appetite, nutritional status, as well as pulmonary function [158]. Van Biervliet et al. show that a high-dose (5 mg zinc/kg/day) supplementation was associated with beneficial effects on growth and pulmonary function [159]. ESPGHAN guidelines suggest zinc supplementation for people with CF who are at risk of a zinc insufficiency [160]. At present, despite contradictory findings, many researchers believe that the intake of 30 mg/day of zinc reduces the number of days of antibiotics used to treat respiratory tract infections in these children [160].

## 8. Pancreatic Enzyme Replacement Therapy

The pancreatic enzyme replacement therapy (PERT) is a standard of care in the management of CF, in order to maintain an adequate nutritional status. PERT is required by 80–90% of CF patients [161]. PERT means the oral administration and delivery of exogenous pancreatic enzymes, especially lipase, amylase and protease, to the lumen of the duodenum, in order to ensure the digestion of fat and protein [8].

Pancreatic enzymes are given as enteric-coated microtablets or microspheres, thus protecting them from gastric acid degradation and ensuring their activation in the alkaline environment of the duodenum (pH > 5.0–5.5) [8]. Similarly, alkalizing the proximal small intestine through the administration of an H2 antagonist or proton pump inhibitor may improve the effectiveness of PERT [10]. Owing to the documented association between the pancreatic phenotype and genotype, PERT should be initiated in patients with two CFTR mutations known to cause PI [130]. Regardless of the CFTR mutation, PERT should be started also in case of unequivocal signs or symptoms of malabsorption, and in patients with a laboratory evidence of PI, with a fecal elastase-1 lower than 200 µg/g in a formed stool, or in those younger than six months with a fecal coefficient of fat absorption lower than 85% [43].

Multiple factors affect the PERT efficacy, such as the degree of PI, state of the disease, hyperacidity of the small bowel or delayed gastric emptying, diet composition and patient compliance [8,43]. Thus, despite multiple guidelines that have been published, PERT doses need to be individualized on the basis of patient age, body weight and grams of fat ingested [10]. The recommended PERT doses in the last ESPEN-ESPGHAN guidelines are consistent with those of the American CF Foundation and with those from another recent consensus [10]. For children younger than 12 months, 2000–4000 units of lipase per 120 mL of milk (breast milk or standard formula) or 2000 units per gram of fat eaten are recommended; for children aged one to four years old, the doses of pancreatic enzymes recommended are 2000–4000 lipase units for gram of fat eaten; children older than four years may receive 500 units of lipase per kilogram of body weight per meal [8,10,76,130,161,162,163]. The dose should gradually be titrated, with a maximum daily dose of 10,000 lipase units per kilogram of body weight per day, or 1000–2500 units per kilogram per meal, or 2000–4000 units per gram of fat eaten [8,10,76,130,161,162,163].

Adverse side effects of PERT are uncommon. However, an increase in enzyme doses beyond the upper recommended limit has been linked to stenotic fibrosing colonopathy and other side effects, such as abdominal bloating, cramping, fullness and nausea [8,130,161].

The response to PERT should be routinely assessed, checking the nutrition status and weight gain, the consistency and colour of stools and the presence of gastrointestinal symptoms [8]. The adequacy of a PERT treatment should be assessed at every clinic visit for infants and every 3 months for older children and adolescents [10].

## 9. Intensified Feeding Intervention

A strict nutritional assessment is crucial to determine when to intensify the nutrition intervention. As previously discussed, a single evaluation of the weight-for-length or BMI percentiles, along with a decline or flattening of the longitudinal growth trajectory, are the currently recognized predictors of nutritional risks for children younger than two or for those between two and 18 years old [10,71]. According to ESPEN/ESPGHAN guidelines [10], nutritional support with diet modification and/or oral nutritional supplementation (ONS) could be initiated with the weight-for-length between the 10th and 50th percentile in children under two years of age. In those between two and 18 years old, the intensification of the nutritional support is required in case of:

- A BMI percentile between the 10th and 50th percentile; or
- Weight loss in the previous 2 to 4 months; or
- No weight gain in the last 2 months.

If there is persistent undernutrition, that is pointed out in children under two years by a weight-for-length percentile under the 10th, and in children aged two–18 by a BMI percentile under the 10th or a weight loss of two percentile points since the last control and stunting of growth, an enteral feeding tube is recommended [10]. The CFF stresses the importance of an early achievement of the nutritional goals, especially by two years of age [130]. According to the CFF guidelines, the evaluation of children at risk includes the calculation of the normal average weight gain. If not achieved, an increase of the caloric intake should be considered through a diet modification and/or oral nutritional supplementation (ONS) [130].

Along with the anthropometric parameters, other predictors of nutritional risk [10,130], which can prelude an intensified nutrition intervention, are:

- Metabolic issues or factors that increase the energy expenditure: recurrent or chronic pulmonary infections or exacerbations, impaired glucose tolerance, salt depletion, zinc deficiency, periods of rapid growth;
- Factors that contribute to malnutrition: problems with doses or adherence to the PERT administration, the need of PPI therapy;
- Factors that decrease the energy intake: gastro-esophageal reflux disease, constipation, celiac or Crohn’s disease, poor appetite and depression.

Taking into account these considerations, the introduction of an enteral feeding tube or parenteral feeding should be considered [164] if a progressive decrease in weight occurs despite:

- Modification in diet in order to improve the caloric intake;
- Control of malabsorption and exclusion of other issues (GERD, CFRLD, other GI diseases);
- Optimal respiratory care;
- Psychologist support.

Data regarding the impact of the new CFTR modulators on the CF outcomes are emerging. Different studies have shown statistically significant improvements, not only in lung function and FEV1, but also in the BMI [165,166]. The fall in pulmonary exacerbations and the lower work of breathing lead to a reduction in the REE and in energy expenditure that enables weight gain and the changes in body composition, with an increase in both fat and lean mass [167,168,169].

Due to these beneficial effects on both respiratory and growth outcomes, it could be presumed that the use of the new modulator drugs could reduce the number of candidates for enteral and parenteral nutrition. However, data in this field are still lacking. Future research should assess the impact of CFTR modulators on nutritional intervention in CF patients.

### 9.1. Oral Nutritional Supplementation

Even if there is conflicting evidence about the efficacy of oral nutritional supplementation (ONS) in weight gain, a high-calorie and fat diet could be not sufficient to improve the nutritional status. Thus, in clinical practice, additional ONS should be considered [10,43]. Products currently available are widely variable for formulations and flavors, to ensure individual preferences and improve compliance [10]. High-energy dense formulations should be given, usually before or after meals or before bedtime [76].

### 9.2. Enteral Nutrition

When the oral caloric intake is not sufficient to reach the anthropometric nutritional goals, supplemental enteral feeding should be initiated to improve growth and nutritional status [10,72,170]. There are various devices, formulas, and timing for feeding enterically; the approach is lastly determined by the patient’s preference and his clinical status [10]. Among the devices for enteral nutrition, gastrostomy tubes are the most frequently used, especially for long term enteral feeding [72]. While feeds may be administered as a bolus during the day, or at a continuous rate overnight, the CFF recommends the latter, in order to encourage the consumption of high-energy meals during the day [10,170].

According to the CFF consensus, the enteral feeding should provide 30–65% of the total estimated calorie and nutrient needs [170]. The Italian Ministry of Health recommends that 50% of the total calories should be consumed during the day and the remaining 50% at night [88]. If comorbidities, such as gastroesophageal reflux disease, gastroparesis or pancreatitis are present, enteral feeds should be introduced directly into the jejunum, via jejunal or gastro-jejunal tubes [72]. In this case, continuous infusions are required. Enteral nutrition can also be administered by a nasogastric tube, which is recommended for short term nutritional supplementation (<3 months), in order to avoid complications, such as tube dislodgement, nasal bleeding or erosion and intestinal perforation [170].

At present, there is no consensus regarding the use of a specific type of formula (polymeric, semi-elemental, elemental) [170]. The polymeric formulas contain whole proteins, oligo- or polysaccharides, medium- or long-chain triglycerides, and are usually iso-osmotic. Elemental formulas are the most hydrolyzed, with small peptides or amino acids instead of proteins; the lipid content is minimal, and are usually hyper-osmotic. Semi-elemental formulas are between elemental and polymeric formulas [171]. In the majority of patients a high-energy polymeric formula (1.5 to 2 Kcal/mL) is at first administered [10]. Clinical experience suggests that isotonic formulas are better tolerated than hypertonic formulas [43]. In case of non-tolerance, an elemental or semi-elemental formula may be preferred [10].

### 9.3. Parenteral Nutrition

Total parenteral nutrition is not routinely advised for nutritional support in patients with CF [10]. Indeed, parenteral nutrition is not recommended for long-term treatment, due to the high risk of complications (such as infection, parenteral-nutrition-associated liver disease), the need for central access for its administration, and the high related cost [10,43]. Nevertheless, parenteral nutrition could be useful for short-term support, in case of contraindications for enteral nutrition (such as intestinal resection, meconium ileus, intestinal failure or short bowel syndrome) and in severely compromised patients enlisted for transplant [10,43,72,76].

## 10. New Therapies and Future Trajectories

In the last decade the therapeutic possibilities towards CF have grown in a consistent way, thanks to a better knowledge of the CFTR structure and genomics and the subsequent pathophysiology leading to disease [10]. The long-term impact of these new therapies on the nutritional domain is largely unknown, mainly because of their relatively recent introduction. Moreover, this theme emerges from numerous studies which are starting to show interesting results and could lead to future trajectories, especially in terms of clinical practice [10]. Starting from a brief examination of the CFTR modulators, we will then extend the discussion on what we know about their role on nutrition and consequently on the microbiome, finishing with the next therapeutic possibility under development.

### 10.1. CFTR Modulators

CFTR modulator therapies are designed to correct the malfunctioning protein made by the CFTR gene. They are mainly represented by small molecules that can be divided into the following categories [172,173]:

Potentiators, which restore or enhance the channel’s open probability;

Correctors, which target misfolding defects and allow protein to travel to the cell surface, enhancing the protein conformational stability during the endoplasmic reticulum folding process;

Stabilizers, which prevent the CFTR’s removal and degradation by anchoring it at the plasma membrane;

Read-through agents, which enable the incorporation of a foreign amino acid in place of the premature termination of the codon of splicing variants during the ribosomal process of translation, making it continue to the normal end of the transcript;

Amplifiers, which increase expression of CFTR mRNA and biosynthesis of the CFTR protein;

Antisense oligonucleotides: correct nonsense mutations and splicing mutations, or deletion mutations (by replacing missing bases).

Notably, many of the drug compounds belonging to the above categories have not reached commercialization, either because they are now undergoing pre-clinical and clinical development, or because of concerns about their safety, tolerability or efficacy. Nevertheless, their high numbers reflect the enormous interest that science and the pharmaceutical industry give to this theme. In Table 1, CFTR modulator therapies that are actually approved and used in the treatment of cystic fibrosis are listed.

Ivacaftor belongs to the potentiator category of CFTR modulators and it was the first one approved in 2012 by FDA and EMA for patients carrying the G551D gating mutation and aged at least six years old [173,174]. Since then, its use has been expanded to many other mutations and to younger ages: to date, 97 CFTR gene mutations are responsive to Kalydeco^®^, and the age from which it is possible to start treatment has been reduced to four months [173,174].

The positive effects of ivacaftor on nutritional aspects consist mainly in improved weight gain and BMI, both in children and adults, as demonstrated in a study during the first 48-week observational period [175]. Differences between the two age cohorts are seen in a longer period, in fact during a subsequent 144-week observational period, only children saw a continuous improvement both in weight and BMI, while adults did not [175]. This difference could be explained by the assumption that adults have reached an optimum in terms of nutritional status, so they cannot improve more [176].

The mechanisms behind these positive changes have not been clearly defined, but it is highly possible that they are primarily linked to an improvement in the CFTR function both in the lungs and in the GI tract, as evidenced by an amelioration in sweat chloride [4]. Concerning the pulmonary effects and their consequences on nutrition, the increased fluidity of the respiratory tract mucus leads to less airway obstruction and fewer pulmonary exacerbations. In fact, it has been demonstrated that a reduction of *Pseudomonas aeruginosa* infections, with improvement of the catabolic state is induced from repeated or prolonged infection [177]: this happens require less work for breathing and consequently in a smaller energy expenditure [175].

The better functioning of CFTR could have other various beneficial effects: by augmenting the fluid flow in the GI tract, it can lead to diminished constipation and SIBBO, thus leading to a better sense of appetite and less intestinal inflammation [175]. A study highlighted an increase in intestinal pH measurements after taking ivacaftor that led to better enzyme functioning, thus leading to an improved absorption of nutrients [177].

Ivacaftor has demonstrated favorable effects also on PI since fecal elastase is normalized after treatment [1]. This explains the good effects, especially on the fat metabolism. Furthermore, glycemic control is known to be influenced by ivacaftor. As insulin secretion improves, its anti-inflammatory effects rise and contribute to a better nutritional status, together with a decline in glycosuria [178,179].

Regarding the other CFTR modulators currently in use, it must first be said that studies on their nutritional effects are not as numerous as the ones made for ivacaftor, mainly for their most recent approval. Lumacaftor is a first-generation corrector, but it also operates as a stabilizer [173]. It has been approved in combination with the potentiator ivacaftor [180] for F508del-homozygous patients aged over 12 years. As it happened for ivacaftor, its use has been extended, both in terms of age and susceptible mutations. Orkambi has favorable effects on pulmonary exacerbations and hospitalizations, and consequently on the improvement in weight gain and BMI [180]. The mechanisms leading to a better nutritional status could be very similar to those observed in the studies conducted for ivacaftor, but more investigations must be carried out to prove it.

The same considerations can be given for the latest CFTR modulators approved. Tezacaftor is a second-generation corrector that has been approved, in combination with ivacaftor [181] for F508del-homozygous or F508del-heterozygous with a residual function mutation in trans patients aged over 12 years. Furthermore, for this combination of modulators there has been an extension, in terms of age and mutations. Compared to ivacaftor alone, the combination tezacaftor/ivacaftor has shown fewer adverse effects and better improvement, in terms of lung function [181]. It is highly probable that the impact on nutrient absorption can be better, as well as on the glucose metabolism. The triple combination ivacaftor/tezacaftor/elexacaftor [182], the last one approved for F508del-homozygous or F508del-heterozygous with a minimal function mutation in trans, has shown important effects in the treatment of CF [182]. In fact, it has a better profile of tolerance and safety, but also better improvements, in terms of pulmonary function and in reduction of exacerbations. Notably, ivacaftor/tezacaftor/elexacaftor was efficacious in patients in whom previous CFTR modulator regimens were ineffective [183]. Given the amount of evidence, it is not difficult to think that a greater effectiveness can also be shown on the gastro-intestinal profile.

Finally, both tezacaftor/ivacaftor and ivacaftor/tezacaftor/elexacaftor will need more studies to better understand the impact they have on the nutritional sphere, given the few studies specifically projected for this field [173]. However, given the accumulating evidence for the previously approved CFTR modulators with a pretty similar mechanism of action, it is likely that the multiple causes underlying the improvement of the nutritional status can be shared between this category of drugs.

Table 2 summarizes the effects of the improvements in the CFTR function.

### 10.2. CFTR Modulators and Impact on the Microbiome

The study of the microbiome has become increasingly central over the years; several strands of research investigate its composition and interactions with many organs and systems, outlining its role in various pathogenetic processes underlying various diseases (gastrointestinal, such as celiac disease, pulmonary disorders, neuropsychiatric problems and many others) [184,185,186]. The role of the microbiome in CF is emerging in the context of the broader concept of the gut–lung axis [187]; the study of the effects of the CFTR-modulating therapies on the microbiome are at their beginning, but some evidence is emerging. A research, although very small, has analyzed the gut microbial communities and gut inflammation using biomarkers, such as fecal calprotectin before and after starting ivacaftor. An increase in beneficial bacteria (*Anaerostipes*, *Akkermansia* spp.) involved in the mucosal protection and a decrease in fecal calprotectin have been noticed, resulting in a decrease in inflammation and an amelioration of the intestinal microbiome [188].

Other studies have focused on the lung microbiome after initiation of the CFTR modulators. Following the beginning of ivacaftor, a greater variability of the microbial population, with a reduction in *P. aeruginosa* species and an increase in endogenous species, especially anaerobes has been observed [189]. Similar results have been seen with Orkambi [190]. These findings may link with a reduction in the exacerbation rate, better lung ventilation and a lower inflammatory status, thus leading to improvements also on the nutritional sphere. Further studies are needed to link these initial discoveries to the impact they may have, in terms of a better nutritional status and their implications in clinical practice.

### 10.3. New Therapies Ongoing: Gene Therapy

A renewed interest in gene therapy has taken place in recent years with the beginning of the genome editing era and especially with the discovery and development of CRISPR-Cas nucleases, whose potential concerns the correction of genetic defects underlying multiple diseases, including CF [190,191]. The CRISPR-Cas nucleases system is based on the use of the Cas9 protein, a “molecular scissor” capable of cutting a target DNA, which can be programmed to make specific modifications to the genome of a cell. Following the cut introduced by Cas9, it is possible to eliminate harmful DNA sequences from the target genome or it is possible to replace sequences. The results from early pre-clinical and clinical studies suggest the use of this technology for the treatment of numerous genetic diseases, including CF [190,191], but much more work is needed in order to overcome several problems, such as the high numbers of CF mutations.

Another approach is gene complementation, in which an additional copy of the wild-type CFTR is delivered to the cells homozygous for CFTR mutations. This strategy can have some advantages, compared to gene repair: potentially, a single pharmaceutical entity could treat all classes of CF mutations, and so far, all CF patients [192].

Whenever such therapies will be implemented in clinical practice, tailored studies towards the nutritional field must be designed and conducted to understand the implications they may have. It is not difficult to think that the results could be extremely positive, as the studies on other drug compounds already available (i.e., CFTR modulators), are already beginning to show.

## 11. Conclusions

Clinical nutrition in CF patients has become very important over the years. Knowledge of the various micro- and macro-nutrient needs, enteral and parenteral nutrition, PERT, CFTR modulator therapies and their practical implementation in clinical experience has been demonstrated to have positive effects on the pulmonary function and also on the other organs and systems. All of these findings are definitely improving the quality of life of thousands of people.

In recent years, there has been a shift over time, from a focus on the pulmonary components of CF care to an emphasis on nutritional care of CF, that is such an integral part of CF treatment. The nutrition support goal in CF care includes achieving an optimal nutritional status, maintenance of the optimal pulmonary function and support growth and puberty development in children. Regarding novel therapeutics for CF, several challenges need to be overcome with the development of drugs that restore the CFTR function in all people with CF, regardless of CFTR genotype, in order to prevent or delay irreversible damage of the lungs and compromisation of the nutritional status. The aim is to transform CF from a fatal disease to a treatable chronic disease through highly efficacious CFTR-directed medicines and specialized multidisciplinary care, as a model for other chronic disorders and genetic diseases.

## Figures and Tables

**Table 1:** CFTR modulators and their impact on nutritional parameters.

Year of Approval by FDA and EMA	CFTR Modulator	Impacts on Nutritional Parameters
2012	Ivacaftor	Improvements in weight gain and BMI (weight and BMI are often primary outcomes that are investigated in the studies concerning the approval of CFTR modulators) Improvements in lung function→fewer exacerbations→better nutritional status, along with better sense of appetite Pancreatic wellness (normalization of fecal elastase), better glycemic control, better absorption of nutrients→better quality of life
2015	Lumacaftor/Ivacaftor	
2018	Tezacaftor/Ivacaftor	
2019	Elexacaftor/Tezacaftor/Ivacaftor	

**Table 2:** Effects of the improvements in the CFTR function.

Improvements in *CFTR* Function	
Pulmonary effects	↑ fluidity in respiratory tract mucus, ↓ airways obstruction, ↓ pulmonary exacerbations
	↓ infectious episodes, ↓ work of breathing, ↓ energy expenditure, ↑ catabolic and nutritional status
Gastro-intestinal effects	↑ fluid flow, ↓ constipation, ↓ small intestinal bacterial overgrowth
	↓ intestinal inflammation, ↑ sense of appetite, ↑ nutritional status
	↑ pH, ↑ enzymatic functioning, ↑ nutrients absorption
	↑ pancreatic wellness (normalization of fecal elastase)
Glycemic control	↑ insuline secretion, ↑ insulin anti-inflammatory effects ↓ glycosuria, ↑ nutritional status
Overall effects	↑ weight and BMI, ↑ pulmonary function, ↓ exacerbations, ↑ quality of life

↑, increase; ↓, decrease.

## Data Availability

Not applicable.

## References

[1] Houwen R.H.J., van der Woerd W.L., Slae M., Wilschanski M. (2017). Effects of new and emerging therapies on gastrointestinal outcomes in cystic fibrosis. Curr. Opin. Pulm. Med..

[2] Hoen A.G., Li J., Moulton L.A., O’Toole G.A., Housman M.L., Koestler D.C., Guill M.F., Moore J.H., Hibberd P.L., Morrison H.G. (2015). Associations between Gut Microbial Colonization in Early Life and Respiratory Outcomes in Cystic Fibrosis. J. Pediatr..

[3] Van Biervliet S., Hauser B., Verhulst S., Stepman H., Delanghe J., Warzee J.-P., Pot B., Vandewiele T., Wilschanski M. (2018). Probiotics in cystic fibrosis patients: A double blind crossover placebo controlled study: Pilot study from the ESPGHAN Working Group on Pancreas/CF. Clin. Nutr. ESPEN.

[4] Ramsey B.W., Davies J., McElvaney N.G., Tullis E., Bell S.C., Dřevínek P., Griese M., McKone E.F., Wainwright C.E., Konstan M.W. (2011). A CFTR potentiator in patients with cystic fibrosis and the G551D mutation. N. Engl. J. Med..

[5] Rafeeq M.M., Murad H.A.S. (2017). Cystic fibrosis: Current therapeutic targets and future approaches. J. Transl. Med..

[6] Gelfond D., Borowitz D. (2013). Gastrointestinal complications of cystic fibrosis. Clin. Gastroenterol. Hepatol..

[7] Uc A., Fishman D.S. (2017). Pancreatic Disorders. Pediatr. Clin. N. Am..

[8] Schindler T., Michel S., Wilson A.W.M. (2015). Nutrition Management of Cystic Fibrosis in the 21st Century. Nutr. Clin. Pract..

[9] Li L., Somerset S. (2014). Digestive system dysfunction in cystic fibrosis: Challenges for nutrition therapy. Dig. Liver Dis..

[10] Turck D., Braegger C.P., Colombo C., Declercq D., Morton A., Pancheva R., Robberecht E., Stern M., Strandvik B., Wolfe S. (2016). ESPEN-ESPGHAN-ECFS guidelines on nutrition care for infants, children, and adults with cystic fibrosis. Clin. Nutr..

[11] Solomon M., Bozic M., Mascarenhas M.R. (2016). Nutritional Issues in Cystic Fibrosis. Clin. Chest Med..

[12] Mauch R.M., Kmit A.H.P., de Lima Marson F.A., Levy C.E., de Azevedo Barros-Filho A., Ribeiro J.D. (2016). Association of growth and nutritional parameters with pulmonary function in cystic fibrosis: A literature review. Rev. Paul. Pediatr..

[13] Collins S. (2018). Nutritional management of cystic fibrosis—An update for the 21st century. Paediatr. Respir. Rev..

[14] Wolfe S.P., Collins C. (2017). The changing face of nutrition in cystic fibrosis. J. Cyst. Fibros..

[15] Farrell P.M., Kosorok M.R., Rock M.J., Laxova A., Zeng L., Lai H.-C., Hoffman G., Laessig R.H., Splaingard M.L., the Wisconsin Cystic Fibrosis Neonatal Screening Study Group (2001). Early diagnosis of cystic fibrosis through neonatal screening prevents severe malnutrition and improves long-term growth. Wisconsin Cystic Fibrosis Neonatal Screening Study Group. Pediatrics.

[16] Gaskin K., Gurwitz D., Durie P., Corey M., Levison H., Forstner G. (1982). Improved respiratory prognosis in patients with cystic fibrosis with normal fat absorption. J. Pediatr..

[17] Kraemer R., Rüdeberg A., Hadorn B., Rossi E. (1978). Relative underweight in cystic fibrosis and its prognostic value. Acta Paediatr Scand..

[18] de Miranda Chaves C.R.M., de Britto J.A.A., de Oliveira C.Q., Gomes M.M., da Cunha A.L.P. (2009). Association between nutritional status measurements and pulmonary function in children and adolescents with cystic fibrosis. J. Bras. Pneumol..

[19] Konstan M.W., Butler S.M., Wohl M.E.B., Stoddard M., Matousek R., Wagener J.S., Johnson C.A., Morgan W.J. (2003). Growth and nutritional indexes in early life predict pulmonary function in cystic fibrosis. J. Pediatr..

[20] Sanders D.B., Fink A., Mayer-Hamblett N., Schechter M.S., Sawicki G.S., Rosenfeld M., Flume P.A., Morgan W.J. (2015). Early Life Growth Trajectories in Cystic Fibrosis are Associated with Pulmonary Function at Age 6 Years. J. Pediatr..

[21] Yen E.H., Quinton H., Borowitz D. (2013). Better nutritional status in early childhood is associated with improved clinical outcomes and survival in patients with cystic fibrosis. J. Pediatr..

[22] Ashkenazi M., Nathan N., Sarouk I., Bar Aluma B.E., Dagan A., Bezalel Y., Keler S., Vilozni D., Efrati O. (2019). Nutritional Status in Childhood as a Prognostic Factor in Patients with Cystic Fibrosis. Lung.

[23] Buchdahl R.M., Fulleylove C., Marchant J.L., Warner J.O., Brueton M.J. (1989). Energy and nutrient intakes in cystic fibrosis. Arch. Dis. Child..

[24] Freedman S.D., Blanco P.G., Zaman M.M., Shea J.C., Ollero M., Hopper I.K., Weed D.A., Gelrud A., Regan M.M., Laposata M. (2004). Association of cystic fibrosis with abnormalities in fatty acid metabolism. N. Engl. J. Med..

[25] Bronstein M., Sokol R., Abman S., Chatfield B., Hammond K., Hambidge K., Stall C., Accurso F. (1992). Pancreatic insufficiency, growth, and nutrition in infants identified by newborn screening as having cystic fibrosis. J. Pediatr..

[26] Fridge J.L., Conrad C., Gerson L., Castillo R.O., Cox K. (2007). Risk factors for small bowel bacterial overgrowth in cystic fibrosis. J. Pediatr. Gastroenterol. Nutr..

[27] Lee J.M., Leach S.T., Katz T., Day A.S., Jaffe A., Ooi C.Y. (2012). Update of faecal markers of inflammation in children with cystic fibrosis. Mediat. Inflamm..

[28] Kopelman H., Corey M., Gaskin K., Durie P., Weizman Z., Forstner G. (1988). Impaired chloride secretion, as well as bicarbonate secretion, underlies the fluid secretory defect in the cystic fibrosis pancreas. Gastroenterology.

[29] Singh V.K., Schwarzenberg S.J. (2017). Pancreatic insufficiency in Cystic Fibrosis. J. Cyst. Fibros..

[30] Augarten A., Tov A.B., Madgar I., Barak A., Akons H., Laufer J., Efrati O., Aviram M., Bentur L., Blau H. (2008). The changing face of the exocrine pancreas in cystic fibrosis: The correlation between pancreatic status, pancreatitis and cystic fibrosis genotype. Eur. J. Gastroenterol. Hepatol..

[31] Pratha V.S., Hogan D.L., Martensson B.A., Bernard J., Zhou R., Isenberg J.I. (2000). Identification of transport abnormalities in duodenal mucosa and duodenal enterocytes from patients with cystic fibrosis. Gastroenterology.

[32] Trang T., Chan J., Graham D.Y. (2014). Pancreatic enzyme replacement therapy for pancreatic exocrine insufficiency in the 21(st) century. World J. Gastroenterol..

[33] Zentler-Munro P.L., Fine D.R., Fitzpatrick W.J., Northfield T.C. (1984). Effect of intrajejunal acidity on lipid digestion and aqueous solubilisation of bile acids and lipids in health, using a new simple method of lipase inactivation. Gut.

[34] Gelfond D., Ma C., Semler J., Borowitz D. (2013). Intestinal pH and gastrointestinal transit profiles in cystic fibrosis patients measured by wireless motility capsule. Dig. Dis. Sci..

[35] Borowitz D., Durie P.R., Clarke L.L., Werlin S.L., Taylor C.J., Semler J., De Lisle R.C., Lewindon P., Lichtman S.M., Sinaasappel M. (2005). Gastrointestinal outcomes and confounders in cystic fibrosis. J. Pediatr. Gastroenterol. Nutr..

[36] Jakab R.L., Collaco A.M., Ameen N.A. (2013). Characterization of CFTR High Expresser cells in the intestine. Am. J. Physiol. Gastrointest. Liver Physiol..

[37] De Lisle R.C., Borowitz D. (2013). The cystic fibrosis intestine. Cold Spring Harb. Perspect. Med..

[38] De Lisle R.C., Roach E., Jansson K. (2007). Effects of laxative and N-acetylcysteine on mucus accumulation, bacterial load, transit, and inflammation in the cystic fibrosis mouse small intestine. Am. J. Physiol. Gastrointest. Liver Physiol..

[39] Dorsey J., Gonska T. (2017). Bacterial overgrowth, dysbiosis, inflammation, and dysmotility in the Cystic Fibrosis intestine. J. Cyst. Fibros..

[40] De Lisle R.C., Meldi L., Mueller R. (2012). Intestinal smooth muscle dysfunction develops postnatally in cystic fibrosis mice. J. Pediatr. Gastroenterol. Nutr..

[41] de Lisle R.C., Sewell R., Meldi L. (2010). Enteric circular muscle dysfunction in the cystic fibrosis mouse small intestine. Neurogastroenterol. Motil..

[42] Palm K., Sawicki G., Rosen R. (2012). The impact of reflux burden on Pseudomonas positivity in children with cystic fibrosis. Pediatr. Pulmonol..

[43] Ratchford T.L., Teckman J.H., Patel D.R. (2018). Gastrointestinal pathophysiology and nutrition in cystic fibrosis. Expert Rev. Gastroenterol. Hepatol..

[44] Leeuwen L., Magoffin A.K., Fitzgerald D.A., Cipolli M., Gaskin K.J. (2014). Cholestasis and meconium ileus in infants with cystic fibrosis and their clinical outcomes. Arch. Dis. Child..

[45] Drzymała-Czyż S., Dziedzic K., Szwengiel A., Krzyżanowska-Jankowska P., Nowak J.K., Nowicka A., Aringazina R., Drzymała S., Kashirskaya N., Walkowiak J. (2022). Serum bile acids in cystic fibrosis patients—Glycodeoxycholic acid as a potential marker of liver disease. Dig. Liver Dis..

[46] Drzymała-Czyż S., Krzyżanowska-Jankowska P., Dziedzic K., Lisowska A., Kurek S., Goździk-Spychalska J., Kononets V., Woźniak D., Mądry E., Walkowiak J. (2021). Severe Genotype, Pancreatic Insufficiency and Low Dose of Pancreatic Enzymes Associate with Abnormal Serum Sterol Profile in Cystic Fibrosis. Biomolecules.

[47] Gelzo M., Iacotucci P., Sica C., Liguori R., Comegna M., Carnovale V., Dello Russo A., Corso G., Castaldo G. (2020). Influence of pancreatic status on circulating plasma sterols in patients with cystic fibrosis. Clin. Chem. Lab. Med..

[48] Zardini Buzatto A., Abdel Jabar M., Nizami I., Dasouki M., Li L., Abdel Rahman A.M. (2021). Lipidome Alterations Induced by Cystic Fibrosis, CFTR Mutation, and Lung Function. J. Proteome Res..

[49] Nowak J.K., Wojsyk-Banaszak I., Mądry E., Wykrętowicz A., Krzyżanowska P., Drzymała-Czyż S., Nowicka A., Pogorzelski A., Sapiejka E., Skorupa W. (2017). Increased Soluble VCAM-1 and Normal P-Selectin in Cystic Fibrosis: A Cross-Sectional Study. Lung.

[50] Costantini D., Padoan R., Curcio L., Giunta A. (1988). The management of enzymatic therapy in cystic fibrosis patients by an individualized approach. J. Pediatr. Gastroenterol. Nutr..

[51] Steinkamp G., Demmelmair H., Rühl-Bagheri I., von der Hardt H., Koletzko B. (2000). Energy supplements rich in linoleic acid improve body weight and essential fatty acid status of cystic fibrosis patients. J. Pediatr. Gastroenterol. Nutr..

[52] Stark L.J., Bowen A.M., Tyc V.L., Evans S., Passero M.A. (1990). A behavioral approach to increasing calorie consumption in children with cystic fibrosis. J. Pediatr. Psychol..

[53] Shepherd R.W., Holt T.L., Cleghorn G., Ward L.C., Isles A., Francis P. (1988). Short-term nutritional supplementation during management of pulmonary exacerbations in cystic fibrosis: A controlled study, including effects of protein turnover. Am. J. Clin. Nutr..

[54] Daniels L., Davidson G.P., Martin A.J., Pouras T. (1989). Supplemental nasogastric feeding in cystic fibrosis patients during treatment for acute exacerbation of chest disease. Aust. Paediatr. J..

[55] Dalzell A.M., Shepherd R.W., Dean B., Cleghorn G.J., Holt T.L., Francis P.J. (1992). Nutritional rehabilitation in cystic fibrosis: A 5 year follow-up study. J. Pediatr. Gastroenterol. Nutr..

[56] Steinkamp G., von der Hardt H. (1994). Improvement of nutritional status and lung function after long-term nocturnal gastrostomy feedings in cystic fibrosis. J. Pediatr..

[57] Levy L.D., Durie P.R., Pencharz P.B., Corey M.L. (1985). Effects of long-term nutritional rehabilitation on body composition and clinical status in malnourished children and adolescents with cystic fibrosis. J. Pediatr..

[58] Mansell A.L., Andersen J.C., Muttart C.R., Ores C.N., Loeff D.S., Levy J.S., Heird W.C. (1984). Short-term pulmonary effects of total parenteral nutrition in children with cystic fibrosis. J. Pediatr..

[59] Bertrand J.M., Morin C.L., Lasalle R., Patrick J., Coates A.L. (1984). Short-term clinical, nutritional, and functional effects of continuous elemental enteral alimentation in children with cystic fibrosis. J. Pediatr..

[60] Siret D., Bretaudeau G., Branger B., Dabadie A., Dagorne M., David V., de Braekeleer M., Moisan-Petit V., Picherot G., Rault G. (2003). Comparing the clinical evolution of cystic fibrosis screened neonatally to that of cystic fibrosis diagnosed from clinical symptoms: A 10-year retrospective study in a French region (Brittany). Pediatr. Pulmonol..

[61] Sims E.J., Clark A., McCormick J., Mehta G., Connett G., Mehta A., on behalf of the United Kingdom Cystic Fibrosis Database Steering Committee (2007). Cystic fibrosis diagnosed after 2 months of age leads to worse outcomes and requires more therapy. Pediatrics.

[62] Lai H.J., Cheng Y., Cho H., Kosorok M.R., Farrell P.M. (2004). Association between initial disease presentation, lung disease outcomes, and survival in patients with cystic fibrosis. Am. J. Epidemiol..

[63] Lai H.J., Shoff S.M., Farrell P.M., Wisconsin Cystic Fibrosis Neonatal Screening Group (2009). Recovery of birth weight z score within 2 years of diagnosis is positively associated with pulmonary status at 6 years of age in children with cystic fibrosis. Pediatrics.

[64] Davies G. (2020). Does newborn screening improve early lung function in cystic fibrosis?. Paediatr. Respir. Rev..

[65] Collins M.S., Abbott M.A., Wakefield D.B., Lapin C.D., Drapeau G., Hopfer S.M., Greenstein R.M., Cloutier M.M. (2008). Improved pulmonary and growth outcomes in cystic fibrosis by newborn screening. Pediatr. Pulmonol..

[66] Castellani C., Massie J., Sontag M., Southern K.W. (2016). Newborn screening for cystic fibrosis. Lancet Respir. Med..

[67] Gokdemir Y., Karadag B.T. (2021). Sweat Testing and Recent Advances. Front. Pediatr..

[68] Borowitz D., Baker R.D., Stallings V. (2002). Consensus report on nutrition for pediatric patients with cystic fibrosis. J. Pediatr. Gastroenterol. Nutr..

[69] Brownell J.N., Bashaw H., Stallings V.A. (2019). Growth and Nutrition in Cystic Fibrosis. Semin. Respir. Crit. Care Med..

[70] Machogu E., Cao Y., Miller T., Simpson P., Levy H., Quintero D., Goday P.S. (2015). Comparison of WHO and CDC growth charts in predicting pulmonary outcomes in cystic fibrosis. J. Pediatr. Gastroenterol. Nutr..

[71] Stallings V.A., Stark L.J., Robinson K.A., Feranchak A.P., Quinton H., Clinical Practice Guidelines on Growth and Nutrition Subcommittee, Ad Hoc Working Group (2008). Evidence-based practice recommendations for nutrition-related management of children and adults with cystic fibrosis and pancreatic insufficiency: Results of a systematic review. J. Am. Diet. Assoc..

[72] Sullivan J.S., Mascarenhas M.R. (2017). Nutrition: Prevention and management of nutritional failure in Cystic Fibrosis. J. Cyst. Fibros..

[73] Sheikh S., Zemel B.S., Stallings V.A., Rubenstein R.C., Kelly A. (2014). Body composition and pulmonary function in cystic fibrosis. Front. Pediatr..

[74] Kelly A., Moran A. (2013). Update on cystic fibrosis-related diabetes. J. Cyst. Fibros..

[75] Wood L.G., Gibson P.G., Garg M.L. (2005). Circulating markers to assess nutritional therapy in cystic fibrosis. Clin. Chim. Acta.

[76] Sinaasappel M., Stern M., Littlewood J., Wolfe S., Steinkamp G., Heijerman H.G., Robberecht E., Döring G. (2002). Nutrition in patients with cystic fibrosis: A European Consensus. J. Cyst. Fibros..

[77] Bell S.C., Bowerman A.M., Nixon L.E., Macdonald I.A., Elborn J.S., Shale D.J. (2000). Metabolic and inflammatory responses to pulmonary exacerbation in adults with cystic fibrosis. Eur. J. Clin. Investig..

[78] Magoffin A., Allen J.R., McCauley J., Gruca M.A., Peat J., Van Asperen P., Gaskin K. (2008). Longitudinal analysis of resting energy expenditure in patients with cystic fibrosis. J. Pediatr..

[79] Gaskin K.J. (2013). Nutritional care in children with cystic fibrosis: Are our patients becoming better?. Eur. J. Clin. Nutr..

[80] Matel J.L. (2012). Nutritional Management of Cystic Fibrosis. J. Parent. Ent. Nutr..

[81] Deutz N.E.P., Bauer J.M., Barazzoni R., Biolo G., Boirie Y., Bosy-Westphal A., Cederholm T., Cruz-Jentoft A., Krznariç Z., Nair K.S. (2014). Protein intake and exercise for optimal muscle function with aging: Recommendations from the ESPEN Expert Group. Clin. Nutr..

[82] Engelen M.P.K.J., Schroder R., Van der Hoorn K., Deutz N.E.P., Com G. (2012). Use of body mass index percentile to identify fat-free mass depletion in children with cystic fibrosis. Clin. Nutr..

[83] Engelen M.P.K.J., Com G., Deutz N.E.P. (2014). Protein is an important but undervalued macronutrient in the nutritional care of patients with cystic fibrosis. Curr. Opin. Clin. Nutr. Metab. Care.

[84] Matel J.L., Kerner J.A. Nutritional Management of Cystic Fibrosis. https://aspenjournals.onlinelibrary.wiley.com/doi/abs/10.1177/0148607111420156.

[85] Maqbool A., Schall J.I., Garcia-Espana J.F., Zemel B.S., Strandvik B., Stallings V.A. (2008). Serum linoleic acid status as a clinical indicator of essential fatty acid status in children with cystic fibrosis. J. Pediatr. Gastroenterol. Nutr..

[86] Wheelock C.E., Strandvik B. (2020). Abnormal n-6 fatty acid metabolism in cystic fibrosis contributes to pulmonary symptoms. Prostaglandins Leukot. Essent. Fat. Acids.

[87] Hanssens L., Thiebaut I., Lefèvre N., Malfroot A., Knoop C., Duchateau J., Casimir G. (2016). The clinical benefits of long-term supplementation with omega-3 fatty acids in cystic fibrosis patients—A pilot study. Prostaglandins Leukot. Essent. Fat. Acids.

[88] Ministero della Salute Linee Guida per una Corretta Prescrizione di Alimenti a Fini Medici Speciali Erogabili per Soggetti con Fibrosi Cistica. https://www.salute.gov.it/imgs/C_17_pubblicazioni_1438_allegato.pdf.

[89] Rana M., Wong-See D., Katz T., Gaskin K., Whitehead B., Jaffe A., Coakley J., Lochhead A. (2014). Fat-soluble vitamin deficiency in children and adolescents with cystic fibrosis. J. Clin. Pathol..

[90] Nowak I.K., Sobkowiak P., Drzymała-Czyż S., Krzyżanowska-Jankowska P., Sapiejka E., Skorupa W., Pogorzelski A., Nowicka A., Wojsyk-Banaszak I., Kurek S. (2021). Fat-Soluble Vitamin Supplementation Using Liposomes, Cyclodextrins, or Medium-Chain Triglycerides in Cystic Fibrosis: A Randomized Controlled Trial. Nutrients.

[91] Loukou I., Moustaki M., Sardeli O., Plyta M., Katsagoni C.N., Douros K. (2021). Association of vitamin A status with lung function in children and adolescents with cystic fibrosis. Pediatr. Investig..

[92] Greer R.M., Buntain H.M., Lewindon P.J., Wainwright C., Potter J.M., Wong J.C., Francis P.W., Batch J.A., Bell S.C. (2004). Vitamin A levels in patients with CF are influenced by the inflammatory response. J. Cyst. Fibros..

[93] Hakim F., Kerem E., Rivlin J., Bentur L., Stankiewicz H., Bdolach-Abram T., Wilschanski M. (2007). Vitamins A and E and pulmonary exacerbations in patients with cystic fibrosis. J. Pediatr. Gastroenterol. Nutr..

[94] Rust P., Eichler I., Renner S., Elmadfa I. (2000). Long-Term Oral β-Carotene Supplementation in Patients with Cystic Fibrosis—Effects on Antioxidative Status and Pulmonary Function. Ann. Nutr. Metab..

[95] Maqbool A., Stallings V.A. (2008). Update on fat-soluble vitamins in cystic fibrosis. Curr. Opin. Pulm. Med..

[96] Maqbool A., Graham-Maar R.C., Schall J.I., Zemel B.S., Stallings V.A. (2008). Vitamin A intake and elevated serum retinol levels in children and young adults with cystic fibrosis. J. Cyst. Fibros..

[97] Finklea J.D., Grossmann R.E., Tangpricha V. (2011). Vitamin D and Chronic Lung Disease: A Review of Molecular Mechanisms and Clinical Studies. Adv. Nutr. Int. Rev. J..

[98] Quraishi S.A., De Pascale G., Needleman J.S., Nakazawa H., Kaneki M., Bajwa E.K., Camargo C.A., Bhan I. (2015). Effect of Cholecalciferol Supplementation on Vitamin D Status and Cathelicidin Levels in Sepsis: A Randomized, Placebo-Controlled Trial. Crit. Care Med..

[99] Spedding S. (2019). Vitamin D and Human Health.

[100] Chesdachai S., Tangpricha V. (2016). Treatment of vitamin D deficiency in cystic fibrosis. J. Steroid Biochem. Mol. Biol..

[101] Tangpricha V., Kelly A., Stephenson A., Maguiness K., Enders J., Robinson K.A., Marshall B.C., Borowitz D., Cystic Fibrosis Foundation Vitamin D Evidence-Based Review Committee (2012). An Update on the Screening, Diagnosis, Management, and Treatment of Vitamin D Deficiency in Individuals with Cystic Fibrosis: Evidence-Based Recommendations from the Cystic Fibrosis Foundation. J. Clin. Endocrinol. Metab..

[102] Vanstone M.B., Egan M.E., Zhang J.H., Carpenter T.O. (2015). Association between serum 25-hydroxyvitamin D level and pulmonary exacerbations in cystic fibrosis. Pediatr. Pulmonol..

[103] McCauley L.A., Thomas W., Laguna T.A., Regelmann W.E., Moran A., Polgreen L.E. (2014). Vitamin D Deficiency Is Associated with Pulmonary Exacerbations in Children with Cystic Fibrosis. Ann. Am. Thorac. Soc..

[104] Pincikova T., Paquin-Proulx D., Sandberg J.K., Flodström-Tullberg M., Hjelte L. (2017). Clinical impact of vitamin D treatment in cystic fibrosis: A pilot randomized, controlled trial. Eur. J. Clin. Nutr..

[105] Grossmann R.E., Zughaier S.M., Kumari M., Seydafkan S., Lyles R.H., Liu S., Sueblinvong V., Schechter M.S., Stecenko A.A., Ziegler T.R. (2012). Pilot study of vitamin D supplementation in adults with cystic fibrosis pulmonary exacerbation: A randomized, controlled trial. Derm.-Endocrinol..

[106] Sermet-Gaudelus I., Bianchi M.L., Garabédian M., Aris R.M., Morton A., Hardin D.S., Elkin S.L., Compston J.E., Conway S.P., Castanet M. (2011). European cystic fibrosis bone mineralisation guidelines. J. Cyst. Fibros..

[107] Green D., Carson K., Leonard A., Davis J.E., Rosenstein B., Zeitlin P., Mogayzel P. (2008). Current Treatment Recommendations for Correcting Vitamin D Deficiency in Pediatric Patients with Cystic Fibrosis Are Inadequate. J. Pediatr..

[108] Jagannath V.A., Fedorowicz Z., Thaker V., Chang A.B. (2015). Vitamin K supplementation for cystic fibrosis. Cochrane Database Syst. Rev..

[109] Conway S.P., Wolfe S.P., Brownlee K.G., White H., Oldroyd B., Truscott J.G., Harvey J.M., Shearer M.J. (2005). Vitamin K status among children with cystic fibrosis and its relationship to bone mineral density and bone turnover. Pediatrics.

[110] Okano T. (2005). Vitamin D, K and bone mineral density. Clin. Calcium.

[111] Dougherty K.A., Schall J.I., Stallings V.A. (2010). Suboptimal vitamin K status despite supplementation in children and young adults with cystic fibrosis. Am. J. Clin. Nutr..

[112] Beker L.T., Ahrens R.A., Fink R.J., O’Brien M.E., Davidson K.W., Sokoll L.J., Sadowski J.A. (1997). Effect of vitamin K1 supplementation on vitamin K status in cystic fibrosis patients. J. Pediatr. Gastroenterol. Nutr..

[113] Lipman T.O., Shils M.E., Olson J.A., Shike M. (1994). Book Reviews: Modern Nutrition in Health and Disease.

[114] Suskind D.L. (2009). Nutritional deficiencies during normal growth. Pediatr. Clin. N. Am..

[115] Peters S.A., Kelly F.J. (1996). Vitamin E supplementation in cystic fibrosis. J. Pediatr. Gastroenterol. Nutr..

[116] Brigelius-Flohé R. (2009). Vitamin E: The shrew waiting to be tamed. Free Radic. Biol. Med..

[117] Okebukola P.O., Kansra S., Barrett J. (2017). Vitamin E supplementation in people with cystic fibrosis. Cochrane Database Syst. Rev..

[118] Ueda N., Suzuki Y., Rino Y., Takahashi T., Imada T., Takanashi Y., Kuroiwa Y. (2009). Correlation between neurological dysfunction with vitamin E deficiency and gastrectomy. J. Neurol. Sci..

[119] Koscik R.L., Lai H.J., Laxova A., Zaremba K.M., Kosorok M.R., Douglas J.A., Rock M.J., Splaingard M.L., Farrell P.M. (2005). Preventing early, prolonged vitamin E deficiency: An opportunity for better cognitive outcomes via early diagnosis through neonatal screening. J. Pediatr..

[120] Swann I.L., Kendra J.R. (1998). Anaemia, vitamin E deficiency and failure to thrive in an infant. Clin. Lab. Haematol..

[121] Wilfond B.S., Farrell P.M., Laxova A., Mischler E. (1994). Severe hemolytic anemia associated with vitamin E deficiency in infants with cystic fibrosis. Implications for neonatal screening. Clin. Pediatr..

[122] Sommerburg O., Hämmerling S., Schneider S., Okun J., Langhans C.-D., Leutz-Schmidt P., Wielpütz M., Siems W., Gräber S., Mall M. (2021). CFTR Modulator Therapy with Lumacaftor/Ivacaftor Alters Plasma Concentrations of Lipid-Soluble Vitamins A and E in Patients with Cystic Fibrosis. Antioxidants.

[123] Gelfond D., Heltshe S., Ma C., Rowe S.M., Frederick C., Uluer A., Sicilian L., Konstan M., Tullis E., Roach C.R.N. (2017). Impact of CFTR Modulation on Intestinal pH, Motility, and Clinical Outcomes in Patients With Cystic Fibrosis and the G551D Mutation. Clin. Transl. Gastroenterol..

[124] Hacquebard M., Carpentier Y.A. (2005). Vitamin E: Absorption, plasma transport and cell uptake. Curr. Opin. Clin. Nutr. Metab. Care.

[125] Brigelius-Flohé R. (2003). Vitamin E and drug metabolism. Biochem. Biophys. Res. Commun..

[126] Feranchak A.P., Sontag M.K., Wagener J.S., Hammond K.B., Accurso F.J., Sokol R.J. (1999). Prospective, long-term study of fat-soluble vitamin status in children with cystic fibrosis identified by newborn screen. J. Pediatr..

[127] Declercq D., Van Braeckel E., Marchand S., Van Daele S., Van Biervliet S. (2020). Sodium Status and Replacement in Children and Adults Living with Cystic Fibrosis: A Narrative Review. J. Acad. Nutr. Diet..

[128] Coates A.J., Crofton P.M., Marshall T. (2009). Evaluation of salt supplementation in CF infants. J. Cyst. Fibros..

[129] Guimarães E.V., Schettino G.C.M., Camargos P.A.M., Penna F.J. (2012). Prevalence of Hyponatremia at Diagnosis and Factors Associated with the Longitudinal Variation in Serum Sodium Levels in Infants with Cystic Fibrosis. J. Pediatr..

[130] Borowitz D., Robinson K.A., Rosenfeld M., Davis S.D., Sabadosa K.A., Spear S.L., Michel S.H., Parad R.B., White T.B., Cystic Fibrosis Foundation (2009). Cystic Fibrosis Foundation evidence-based guidelines for management of infants with cystic fibrosis. J. Pediatr..

[131] Davison A.G., Snodgrass G.J.A. (1983). Cystic Fibrosis Mimicking Bartter’s Syndrome. Acta Paediatr..

[132] Simopoulos A.P., Lapey A., Boat T.F., Di Santagnese P.A., Bartter F.C. (1971). The Renin-Angiotensin-Aldosterone System in Patients with Cystic Fibrosis of the Pancreas. Pediatr. Res..

[133] Orenstein D.M., Henke K.G., Costill D.L., Doershuk C.F., Lemon P.J., Stern R.C. (1983). Exercise and heat stress in cystic fibrosis patients. Pediatr. Res..

[134] Kriemler S., Wilk B., Schurer W., Wilson W.M., Bar-Or O. (1999). Preventing dehydration in children with cystic fibrosis who exercise in the heat. Med. Sci. Sport. Exerc..

[135] Al-Ghimlas F., Faughnan M.E., Tullis E. (2012). Metabolic alkalosis in adults with stable cystic fibrosis. Open Respir. Med. J..

[136] Bower T.R., Pringle K.C., Soper R.T. (1988). Sodium deficit causing decreased weight gain and metabolic acidosis in infants with ileostomy. J. Pediatr. Surg..

[137] Uijterschout L., Nuijsink M., Hendriks D., Vos R., Brus F. (2014). Iron deficiency occurs frequently in children with cystic fibrosis. Pediat. Pulmonol..

[138] Ehrhardt P., Miller M.G., Littlewood J.M. (1987). Iron deficiency in cystic fibrosis. Arch. Dis. Child..

[139] World Health Organization Anaemia and Iron Deficiency. www.who.int/nutrition/publications/micronutrients/anaemia_iron_deficiency/9789241596107/en/.

[140] Kałużna-Czyż M., Grzybowska-Chlebowczyk U., Woś H., Więcek S. (2018). Serum Hepcidin Level as a Marker of Iron Status in Children with Cystic Fibrosis. Mediat. Inflamm..

[141] Uijterschout L., Swinkels D.W., Akkermans M.D., Zandstra T., Nuijsink M., Hendriks D., Hudig C., Tjalsma H., Vos R., van Goudoever J.B. (2014). The value of soluble transferrin receptor and hepcidin in the assessment of iron status in children with cystic fibrosis. J. Cyst. Fibros..

[142] Sands D., Mielus M., Umławska W., Lipowicz A., Oralewska B., Walkowiak J. (2015). Evaluation of factors related to bone disease in Polish children and adolescents with cystic fibrosis. Adv. Med. Sci..

[143] Grey V., Atkinson S., Drury D., Casey L., Ferland G., Gundberg C., Lands L.C. (2008). Prevalence of Low Bone Mass and Deficiencies of Vitamins D and K in Pediatric Patients with Cystic Fibrosis from 3 Canadian Centers. Pediatrics.

[144] Buntain H.M., Greer R.M., Schluter P.J., Wong J.C.H., Batch J.A., Potter J.M., Lewindon P.J., Powell E., Wainwright C.E., Bell S.C. (2004). Bone mineral density in Australian children, adolescents and adults with cystic fibrosis: A controlled cross sectional study. Thorax.

[145] Aris R.M., Merkel P.A., Bachrach L.K., Borowitz D.S., Boyle M.P., Elkin S.L., Guise T.A., Hardin D.S., Haworth C.S., Holick M.F. (2005). Guide to bone health and disease in cystic fibrosis. J. Clin. Endocrinol. Metab..

[146] Gore A.P., Kwon S.H., Stenbit A.E. (2010). A roadmap to the brittle bones of cystic fibrosis. J. Osteoporos..

[147] Haworth C.S. (2010). Impact of cystic fibrosis on bone health. Curr. Opin. Pulm. Med..

[148] Sermet-Gaudelus I., Castanet M., Retsch-Bogart G., Aris R.M. (2009). Update on cystic fibrosis-related bone disease: A special focus on children. Paediatr. Respir. Rev..

[149] Tanumihardjo S.A., Russell R.M., Stephensen C.B., Gannon B.M., Craft N.E., Haskell M.J., Lietz G., Schulze K., Raiten D.J. (2016). Biomarkers of Nutrition for Development (BOND)-Vitamin A Review. J. Nutr..

[150] Lin P.-H., Sermersheim M., Li H., Lee P., Steinberg S., Ma J. (2017). Zinc in Wound Healing Modulation. Nutrients.

[151] Young G.P., Mortimer E.K., Gopalsamy G.L., Alpers D.H., Binder H.J., Manary M.J., Ramakrishna B.S., Brown I.L., Brewer T.G. (2014). Zinc deficiency in children with environmental enteropathy-development of new strategies: Report from an expert workshop. Am. J. Clin. Nutr..

[152] Nussbaum S.R., Carter M.J., Fife C.E., DaVanzo J., Haught R., Nusgart M., Cartwright D. (2018). An Economic Evaluation of the Impact, Cost, and Medicare Policy Implications of Chronic Nonhealing Wounds. Value Health.

[153] Ibs K.-H., Rink L. (2003). Zinc-altered immune function. J. Nutr..

[154] Salgueiro M.J., Zubillaga M.B., Lysionek A.E., Caro R.A., Weill R., Boccio J.R. (2002). The role of zinc in the growth and development of children. Nutrition.

[155] Prasad A.S. (1985). Clinical manifestations of zinc deficiency. Annu. Rev. Nutr..

[156] Ataee P., Najafi M., Gharagozlou M., Aflatounian M., Mahmoudi M., Khodadad A., Farahmand F., Motamed F., Fallahi G.H., Kalantari N. (2014). Effect of supplementary zinc on body mass index, pulmonary function and hospitalization in children with cystic fibrosis. Turk. J. Pediatr..

[157] Maqbool A., Schall J.I., Zemel B.S., Felipe Garcia-Espana J., Stallings V.A. (2006). Plasma Zinc and Growth Status in Preadolescent Children with Cystic Fibrosis. J. Pediatr. Gastroenterol. Nutr..

[158] Van Biervliet S., Van Biervliet J.-P., Robberecht E., Taylor C. (2009). Importance of zinc in cystic fibrosis patients. Curr. Pediatr. Rev..

[159] Van Biervliet S., Vande Velde S., Van Biervliet J.P., Robberecht E. (2008). The effect of zinc supplements in cystic fibrosis patients. Ann. Nutr. Metab..

[160] Abdulhamid I., Beck F.W.J., Millard S., Chen X., Prasad A. (2008). Effect of zinc supplementation on respiratory tract infections in children with cystic fibrosis. Pediatr. Pulmonol..

[161] Berry A.J. (2014). Pancreatic enzyme replacement therapy during pancreatic insufficiency. Nutr. Clin. Pract..

[162] Sermet-Gaudelus I., Mayell S.J., Southern K.W., European Cystic Finrosis Society (ECFS), Neonatal Screening Working Group (2010). Guidelines on the early management of infants diagnosed with cystic fibrosis following newborn screening. J. Cyst. Fibros..

[163] Haupt M.E., Kwasny M.J., Schechter M.S., McColley S.A. (2014). Pancreatic enzyme replacement therapy dosing and nutritional outcomes in children with cystic fibrosis. J. Pediatr..

[164] (2020). Clinical Guidelines: Care of Children with Cystic Fibrosis. https://www.rbht.nhs.uk/childrencf.

[165] De Boeck K., Munck A., Walker S., Faro A., Hiatt P., Gilmartin G., Higgins M. (2014). Efficacy and safety of ivacaftor in patients with cystic fibrosis and a non-G551D gating mutation. J. Cyst. Fibros..

[166] Dryden C., Wilkinson J., Young D., Brooker R.J., Scottish Paediatric Cystic Fibrosis Managed Clinical Network (SPCFMCN) (2018). The impact of 12 months treatment with ivacaftor on Scottish paediatric patients with cystic fibrosis with the G551D mutation: A review. Arch. Dis. Child..

[167] Stallings V.A., Sainath N., Oberle M., Bertolaso C., Schall J.I. (2018). Energy Balance and Mechanisms of Weight Gain with Ivacaftor Treatment of Cystic Fibrosis Gating Mutations. J. Pediatr..

[168] Burghard M., Berkers G., Ghijsen S., Hollander-Kraaijeveld F.M., de Winter-de Groot K.M., van der Ent C.K., Heijerman H.G.M., Takken T., Hulzebos H.J. (2020). Long-term effects of ivacaftor on nonpulmonary outcomes in individuals with cystic fibrosis, heterozygous for a S1251N mutation. Pediatr. Pulmonol..

[169] Karb D.B., Cummings L.C. (2021). The Intestinal Microbiome and Cystic Fibrosis Transmembrane Conductance Regulator Modulators: Emerging Themes in the Management of Gastrointestinal Manifestations of Cystic Fibrosis. Curr. Gastroenterol. Rep..

[170] Schwarzenberg S.J., Hempstead S.E., McDonald C.M., Powers S.W., Wooldridge J., Blair S., Freedman S., Harrington E., Murphy P.J., Palmer L. (2016). Enteral tube feeding for individuals with cystic fibrosis: Cystic Fibrosis Foundation evidence-informed guidelines. J. Cyst. Fibros..

[171] Erskine J.M., Lingard C., Sontag M. (2007). Update on enteral nutrition support for cystic fibrosis. Nutr. Clin. Pract..

[172] Lopes-Pacheco M. (2019). CFTR Modulators: The Changing Face of Cystic Fibrosis in the Era of Precision Medicine. Front. Pharmacol..

[173] Meoli A., Eickmeier O., Pisi G., Fainardi V., Zielen S., Esposito S. (2022). Impact of CFTR Modulators on the Impaired Function of Phagocytes in Cystic Fibrosis Lung Disease. Int. J. Mol. Sci..

[174] Food and Drug Administration Highlights of Prescribing Information. Kalydeco. https://www.accessdata.fda.gov/drugsatfda_docs/label/2020/203188s034,207925s013lbl.pdf.

[175] Borowitz D., Lubarsky B., Wilschanski M., Munck A., Gelfond D., Bodewes F., Schwarzenberg S.J. (2016). Nutritional Status Improved in Cystic Fibrosis Patients with the G551D Mutation after Treatment with Ivacaftor. Dig. Dis. Sci..

[176] McKone E.F., Borowitz D., Drevinek P., Griese M., Konstan M.W., Wainwright C., Ratjen F., Sermet-Gaudelus I., Plant B., Munck A. (2014). Long-term safety and efficacy of ivacaftor in patients with cystic fibrosis who have the Gly551Asp-CFTR mutation: A phase 3, open-label extension study (PERSIST). Lancet Respir. Med..

[177] Rowe S.M., Heltshe S.L., Gonska T., Donaldson S.H., Borowitz D., Gelfond D., Sagel S.D., Khan U., Mayer-Hamblett N., Van Dalfsen J.M. (2014). Clinical mechanism of the cystic fibrosis transmembrane conductance regulator potentiator ivacaftor in G551D-mediated cystic fibrosis. Am. J. Respir. Crit. Care Med..

[178] Bellin M.D., Laguna T., Leschyshyn J., Regelmann W., Dunitz J., Billings J., Moran A. (2013). Insulin secretion improves in cystic fibrosis following ivacaftor correction of CFTR: A small pilot study. Pediatr. Diabetes.

[179] Hayes D., McCoy K.S., Sheikh S.I. (2014). Resolution of cystic fibrosis-related diabetes with ivacaftor therapy. Am. J. Respir. Crit. Care Med..

[180] Konstan M.W., McKone E.F., Moss R.B., Marigowda G., Tian S., Waltz D., Huang X., Lubarsky B., Rubin J., Millar S.J. (2017). Assessment of safety and efficacy of long-term treatment with combination lumacaftor and ivacaftor therapy in patients with cystic fibrosis homozygous for the F508del-CFTR mutation (PROGRESS): A phase 3, extension study. Lancet Resp. Med..

[181] Rowe S.M., Daines C., Ringshausen F.C., Kerem E., Wilson J., Tullis E., Nair N., Simard C., Han L., Ingenito E.P. (2017). Tezacaftor-Ivacaftor in Residual-Function Heterozygotes with Cystic Fibrosis. N. Engl. J. Med..

[182] Keating D., Marigowda G., Burr L.D., Daines C., Mall M.A., McKone E.F., Ramsey B.W., Rowe S.M., Sass L.A., Tullis E. (2018). VX-445-Tezacaftor-Ivacaftor in Patients with Cystic Fibrosis and One or Two Phe508del Alleles. N. Engl. J. Med..

[183] Middleton P.G., Mall M.A., Dřevínek P., Lands L.C., McKone E.F., Polineni D., Ramsey B.W., Taylor-Cousar J.L., Tullis E., Vermeulen F. (2019). Elexacaftor-Tezacaftor-Ivacaftor for Cystic Fibrosis with a Single Phe508del Allele. N. Engl. J. Med..

[184] Pecora F., Persico F., Gismondi P., Fornaroli F., Iuliano S., de’Angelis G.L., Esposito S. (2020). Gut Microbiota in Celiac Disease: Is There Any Role for Probiotics?. Front. Immunol..

[185] Frati F., Salvatori C., Incorvaia C., Bellucci A., Di Cara G., Marcucci F., Esposito S. (2018). The Role of the Microbiome in Asthma: The Gut–Lung Axis. Int. J. Mol. Sci..

[186] Fattorusso A., Di Genova L., Dell’Isola G., Mencaroni E., Esposito S. (2019). Autism Spectrum Disorders and the Gut Microbiota. Nutrients.

[187] Esposito S., Testa I., Mariotti Zani E., Cunico D., Torelli L., Grandinetti R., Fainardi V., Pisi G., Principi N. (2022). Probiotics Administration in Cystic Fibrosis: What Is the Evidence?. Nutrients.

[188] Ooi C.Y., Syed S.A., Rossi L., Garg M., Needham B., Avolio J., Young K., Surette M.G., Gonska T. (2018). Impact of CFTR modulation with Ivacaftor on Gut Microbiota and Intestinal Inflammation. Sci. Rep..

[189] Bernarde C., Keravec M., Mounier J., Gouriou S., Rault G., Férec C., Barbier G., Héry-Arnaud G. (2015). Impact of the CFTR-potentiator ivacaftor on airway microbiota in cystic fibrosis patients carrying a G551D mutation. PLoS ONE.

[190] Neerincx A.H., Whiteson K., Phan J.L., Brinkman P., Abdel-Aziz M.I., Weersink E.J., Altenburg J., Majoor C.J., der Zee A.H.M.-V., Bos L.D. (2021). Lumacaftor/ivacaftor changes the lung microbiome and metabolome in cystic fibrosis patients. ERJ Open. Res..

[191] Maule G., Arosio D., Cereseto A. (2020). Gene Therapy for Cystic Fibrosis: Progress and Challenges of Genome Editing. Int. J. Mol. Sci..

[192] Gill D.R., Hyde S.C. (2014). Delivery of genes into the CF airway. Thorax.

